# Path Following, Obstacle Detection and Obstacle Avoidance for Thrusted Underwater Snake Robots

**DOI:** 10.3389/frobt.2019.00057

**Published:** 2019-07-23

**Authors:** Eleni Kelasidi, Signe Moe, Kristin. Y. Pettersen, Anna M. Kohl, Pål Liljebäck, Jan Tommy Gravdahl

**Affiliations:** ^1^Department of Engineering Cybernetics, Centre for Autonomous Marine Operations and Systems, Norwegian University of Science and Technology, Trondheim, Norway; ^2^Department of Seafood Technology, SINTEF Ocean, Trondheim, Norway; ^3^Department of Engineering Cybernetics, Norwegian University of Science and Technology, Trondheim, Norway; ^4^Department of Mathematics and Cybernetics, SINTEF Digital, Oslo, Norway

**Keywords:** underwater snake robots, energy efficiency, thrusted USR, path following, obstacle detection and avoidance

## Abstract

The use of unmanned underwater vehicles is steadily increasing for a variety of applications such as mapping, monitoring, inspection and intervention within several research fields and industries, e.g., oceanography, marine biology, military, and oil and gas. Particularly interesting types of unmanned underwater vehicles are bio-inspired robots such as underwater snake robots (USRs). Due to their flexible and slender body, these versatile robots are highly maneuverable and have better access capabilities than more conventional remotely operated vehicles (ROVs). Moreover, the long and slender body allows for energy-efficient transit over long distances similar to torpedo-shaped autonomous underwater vehicles (AUVs). In addition, USRs are capable of performing light intervention tasks, thereby providing intervention capabilities which exceed those of AUVs and inspection class ROVs. USRs may also propel themselves using energy-efficient motion patterns inspired by their biological counterparts. They can thereby increase the propulsion efficiency during transit and maneuvering, which is among the great challenges for autonomous underwater vehicles. In this paper, a control system for path following, and algorithms for obstacle detection and avoidance, are presented for a USR with thrusters attached at the tail module. The position of the obstacles is detected using a single camera in the head module of the USR and a developed computer vision algorithm. For the proposed control concept the robot joints are used for directional control while the thrusters are used for forward propulsion. The USR circumvents obstacles by following a circular path around them before converging back to the main straight line path when this is safe. Experimental results that validate the proposed methods are also presented.

## 1. Introduction

Through millions of years of evolution, sea snakes, eels and fish have developed highly efficient motion for propulsion and locomotion. These creatures are able to rapidly change direction in a highly efficient manner (Lighthill, 1970, 1975). Many of them have superior acceleration capabilities, while simultaneously being able to access confined spaces using their flexible bodies. Over the last decades, remotely operated vehicles (ROVs) have been extensively used for subsea inspection, maintenance, and repair operations in the oil and gas industry (Christ and Wernli, 2013). These vehicles rely on being operated by a highly trained human in the loop. In order to make such operations safer and more cost-efficient, there has been an increasing interest in developing intervention AUVs (I-AUVs) (Ridao et al., 2014), underwater snake robots (USRs) (Mclsaac and Ostrowski, 1999; McIsaac and Ostrowski, 2002; Takayama and Hirose, 2002; Wilbur et al., 2002; Crespi et al., 2005; Yamada et al., 2005; Crespi and Ijspeert, 2006; Li et al., 2011; Stefanini et al., 2012; Liljebäck et al., 2014; Kelasidi et al., 2016a,b) and underwater snake robots with thrusters (Sverdrup-Thygeson et al., 2016a,b) as a step toward improved autonomy, dexterity and precision for underwater manipulation tasks. Detailed discussions on different underwater robotic systems such as ROVs, AUVs and bio-inspired robotic systems can be found in Kelasidi et al. (2016a) and Kelasidi et al. (2017b).

**Figure 1:**
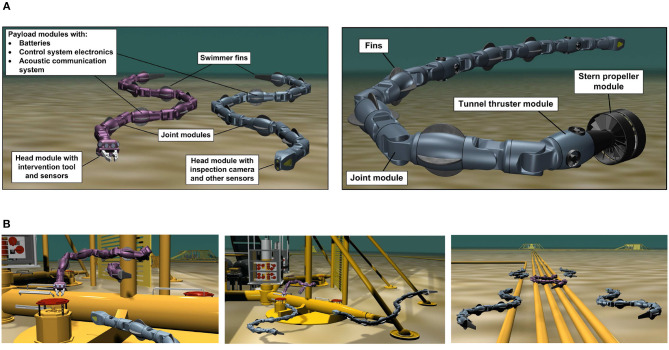
Underwater snake robots are highly flexible, capable of rapid directional changes and can access small and confined spaces. They can perform intervention tasks and efficient transportation for longer range missions. When combined with additional effectors such as thrusters or tail fins, these robots are highly versatile and may be applied for a variety of underwater operations. **(A)** Concept sketches bio-inspired underwater snake robots with additional effectors. **(B)** Next generation of inspection and intervention vehicles for underwater applications.

**Figure 2:**
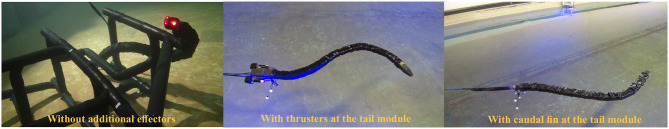
Different configurations of the underwater snake robot Mamba.

Inspired by biological swimming creatures, a novel concept for bio-inspired multi-articulated robotic systems has been illustrated in Figure 1, which combines properties of aquatic animals with state of the art solutions from marine technology. Unlike conventional underwater robotic solutions, the USR is a slender and highly redundant robot, which is able to propel itself forward using body undulations combined with caudal, dorsal and pectoral fins and/or with stern propellers and tunnel thrusters along the body. This provides significant flexibility and increases the maneuverability of the robot for subsea applications, as illustrated in Figure 1 (Kelasidi et al., 2015; Sverdrup-Thygeson et al., 2016b). The modular design of the robot makes it suitable for different applications by simply connecting various modules in different combinations to form various types of vehicles. As illustrated in Figure 1, the robotic system is a dexterous robotic arm which can operate tools and carry out light intervention tasks. In addition, by using either foils or thrusters, it can transit over long distances in a similar manner as a survey AUV, while its flexible and slender body provides the ability to access and operate in restricted areas of subsea structures. The modular design of the robot makes it applicable for different applications depending on the requirement of the task. For instance, a purely bio-inspired solution without using propellers can be considered for applications where limited acoustic noise is required, whereas equipping the robot with thrusters can provide improved maneuverability for inspection and intervention tasks. The use of USRs for such subsea operations introduces several interesting research challenges. Figure 2 presents the first USR equipped with thrusters at the tail module, developed at the Norwegian University of Science and Technology (NTNU) (Liljebäck et al., 2014; Kelasidi et al., 2016b). This robotic platform using thrusters only at the tail module can be considered a special case of the general concept shown in Figure 1, and is a step toward developing the next generation of USRs with additional effectors. The modular snake robot Mamba (Liljebäck et al., 2014) can be equipped with thrusters when operated underwater (Kelasidi et al., 2016b). Mamba with thrusters is a new type of snake-like robot which combines biologically inspired undulatory locomotion with the use of thrusters, and is the test platform considered for all the experimental results presented in this paper.

Obstacle avoidance is a crucial task for numerous robotic systems. For fixed base systems, the robot must avoid self-collisions as well as any objects that might be within its workspace. For floating base robots, such as a USR or a surface ship, the main task is to avoid stationary and dynamic obstacles such as islands/pipelines/other structures and other ships or floating base systems. There exist several path planning algorithms for computing a safe path to avoid obstacles, such as A^⋆^, RRT and HBug (Hernandez et al., 2015). However, these global path planning methods are not suitable for unknown and dynamic environments, and must be complemented by a local guidance system that is able to make the mobile robotic system avoid small, unforeseen, and dynamic obstacles while following the global path. A variety of such local approaches have been proposed, both for the general and maritime case, such as potential fields (Khatib, 1985), dynamic window (Fox et al., 1997; Loe, 2008), velocity obstacles van den Berg et al. (2011); Kuwata et al. (2014), and Tangent/WedgeBug Laubach and Burdick (1999). However, these approaches have several drawbacks. Potential fields may suffer from oscillating behavior and convergence to local minima (Koren and Borenstein, 1991), and the dynamic window approach can be computationally heavy. The velocity obstacle (VO) approach has good mathematical qualities and is computationally simple, but is not straight-forward to implement. However, the main drawback of these methods is the fact that it is not obvious how to combine these collision avoidance methods with existing, commonly used guidance methods for path following such as line-of-sight (LOS) (Fossen, 2011). The Wedgebug algorithm is applied to Mars Rovers and assumes that the rover is modeled as a point robot in a 2D binary environment (i.e., every point in the environment is either contained within an impassable obstacle, or lies in freespace) and that obstacle boundaries block sensing as well as motion. In the approach proposed in this paper, the obstacle boundaries have the possibility to be virtual, which prevents passage into identified unsafe areas without physical obstacles in the way.

In nautical navigation there exists several obstacle avoidance methods which all require some information about the obstacle itself, i.e., position, size and/or velocity. To detect underwater obstacles, one may use sensors such as sonars and cameras (Nicholson and Healey, 2008; Ridao et al., 2014; Mallios et al., 2016). Due to the properties of light propagation under water, acoustics-based navigations methods are often applied. Vision systems decrease the range, but also decrease space and cost and increase the resolution (Bonin-Font et al., 2008). Often, vision systems are based on two cameras, i.e., stereo vision. With such a setup, one can use matching and geometric triangulation to calculate the 3D-position of detected features (Goldberg et al., 2002). The USR Mamba is equipped with a single camera at the head module. However, obstacle avoidance still requires sensing of depth, i.e., the distance between the vision sensor and the obstacle. To achieve this using monocular vision, one must rely on assumptions concerning the scene geometry and vehicle motion (Bhatti, 2008; Lei et al., 2013). In this paper, we have developed a computer vision algorithm to detect potential obstacles along the path of the USR by using a single camera attached at the head module of the robot and reflective markers on the obstacles. The area of the markers is a priori knowledge and can be used to calculate the 3D distance based on the corresponding area in the image similar to Bousaid et al. (2016). Different geometric shapes (i.e., triangle, square and circle). This can be used to classify different types of obstacles. The shape is determined by analyzing the curvature of the shape and counting the number of peaks.

In this paper, we perform experiments to investigate both the path following and obstacle avoidance control problem using the USR Mamba with thrusters (Kelasidi et al., 2016b). The goal of the experiments is to detect potential obstacles along the path and design the USR motion to ensure that the robot can converge to and follow a predefined reference path while avoiding the detected obstacles.

In Kelasidi et al. (2016b), it is suggested that in order to ensure efficient transportation, a USR with thrusters at the tail module should mainly use the thrusters for locomotion, while the multi-articulated body should be used for directional control. Motivated by these results, we propose a motion control strategy for thrusted USRs with an overall goal of investigating its ability to follow a given reference path. Several previous works consider control schemes for locomotion of USRs without thrusters. A comparison of these approaches is presented in Kelasidi et al. (2017b) and Kelasidi et al. (2016a). In addition, a docking approach for thrusted USRs using the joint angles to control the direction of the robot has been presented in Sans-Muntadas et al. (2017).

This paper presents a path following control strategy that is able to make the thrusted USR follow the desired reference path. Furthermore, the developed obstacle detection scheme was successfully applied and combined with a set-based collision avoidance method (Moe and Pettersen, 2016; Kohl et al., 2017). This approach ensures obstacle avoidance when necessary and path following otherwise.

The path following control concept and obstacle avoidance for USRs without thrusters has been investigated in Kohl et al. (2017). Here, both the direction and propulsion are achieved through the undulatory motion of the joints. In this paper, these methods are adapted to USRs with thrusters. In addition, an obstacle detection strategy is presented and combined with the path following and obstacle avoidance methods. The proposed guidance and control strategy and obstacle detection and obstacle avoidance strategy are experimentally validated for USRs with thrusters. To the authors' best knowledge experimental results regarding obstacle detection and avoidance have not been presented in previous literature for thrusted USRs.

This paper is organized as follows: Section 2 presents the experimental setup as well as the guidance and control methods for path following and obstacle avoidance and the obstacle detection algorithm. In section 3, the experimental results are presented and discussed. Conclusions are given in section 4.

## 2. Setup and Control System

In this section, we give a brief description of the thrusted USR Mamba and the experimental setup. Furthermore, we discuss and present how the guidance and control approach proposed earlier for USRs without thrusters are adapted for the experiments with the thrusted Mamba. Finally, the obstacle detection technique adopted in this paper and the set-based obstacle avoidance approach proposed for the thrusted USRs are presented.

### 2.1. Experimental Setup

The underwater snake robot with thrusters at the tail module named Mamba (Figure 2) is basically a self-propelled robotic arm with a slender and flexible body able to access and carry out inspection tasks in confined spaces not accessible by conventional underwater vehicles. Mamba has a modular design and can operate at shallow water depths. For more information about the robot, see Kelasidi et al. (2016a,b). Note that for the thrusted robot it is important to know the amplitude of the applied thruster forces as a function of the particular control input. Initial experiments were performed to obtain the necessary mapping from the thruster inputs *u*_*c*_ to thruster forces *F*_*t*_ for the USR, and the results prove that the relationship is quite linear (Kelasidi et al., 2016b). Another purely bio-inspired configuration of the underwater snake robot Mamba with a passive caudal fin attached at the tail module of the robot (Figure 2) can be advantageous compared to the configuration with thrusters, since it does not produce significant acoustic noise. Moreover, a fin configuration will not perturb the surroundings as much as the thrusters, which is highly relevant for applications such as archaeological investigation of shipwrecks and non-invasive monitoring of marine life. A comparative study of the robot with and without a caudal fin was presented in Kelasidi et al. (2017a). In particular, it was shown that by attaching a passive caudal fin it is possible to double the forward velocity. This significant velocity increase requires a relatively low increase in power consumption, and is achieved with a minimum increase in the complexity of the mechanical design.

**Figure 3:**
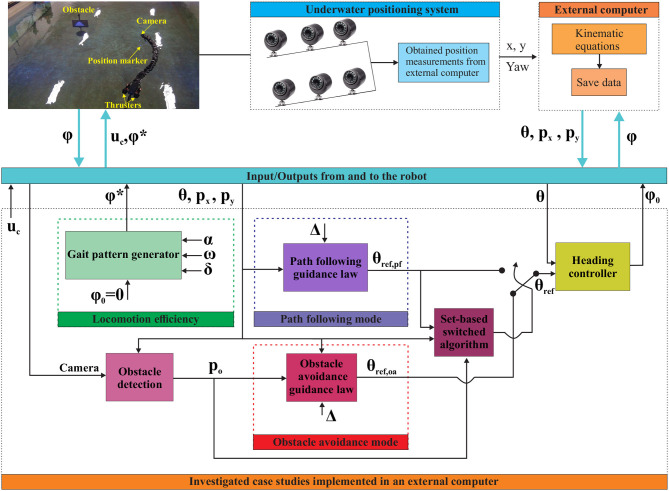
Experimental setup to investigate path following control and obstacle detection and obstacle avoidance using the underwater snake robot Mamba.

The robot considered in the current study consists of 18 joints mounted with a relative orientation of 90 degrees in an alternating fashion to achieve both yaw and pitch motion (Liljebäck et al., 2014). An external skin was used during the experiments in order to achieve an additional water barrier, in addition to making the robot's outer surface more smooth. The experimental setup is illustrated in Figure 3.

The experiments carried out in a basin at the Marine cybernetics laboratory (MC-lab), Trondheim, Norway (MCl, 2018). The basin is 1.5 m deep with a surface area of 40 m × 6.45 m. Six underwater cameras from Qualisys (QUA, 2018) were used to track and log the position and orientation θ of the robot, using a structure with reflective markers attached at the head or tail module. The center of mass (CM) *p*_*x*_, *p*_*y*_ is then calculated using the kinematic equations of the robot (Kelasidi et al., 2016a). As illustrated in Figure 3, the obtained measurements were used to investigate different control challenges for the thrusted underwater snake robot Mamba. During these experiments the joint angles responsible for the vertical (pitch) motion were set to zero degrees to achieve purely horizontal motion. All the algorithms were implemented in an external computer using Labview, and the necessary signals were sent/received to/from the robot through a CAN bus connection through a tether. Figure 3 illustrates three different case studies for USRs with thrusters: (1) Locomotion efficiency studies Kelasidi et al. (2016b), (2) Path following of USR with thrusters, and (3) Switching strategy between path following, and obstacle detection and obstacle avoidance modes developed and experimentally validated in this paper. In the following sections, each part of the case studies illustrated in Figure 3 will be discussed in more detail.

### 2.2. Guidance and Control

The guidance and control system of the USR is illustrated in Figure 3 and the definition of the mathematical symbols are described in Table 1. The guidance system provides a reference θ_ref_ for the orientation of the USR, which the controller attempts to follow by controlling the USR joints. The thrusters are controlled by the input *u*_*c*_ and each joint follows the output from the heading controller ϕ_0_ according to (1). The proposed control strategy assumes that the robot joints are used for directional control, while the propulsion of the robot is given only by the thrusters. It is a decoupled system where the values of the control input *uc* is responsible for controlling the forward velocity of the robot, while the heading controller (1) is responsible for the turning motion of the robot.

**Table 1:** Definition of mathematical terms.

**Symbol**	**Description**
*u*_c_	Thruster inputs
θ	Orientation of the robot
θ_ref_	Reference orientation of the robot
(*x, y*)	Vector of global coordinates of the CM of links
(*p*_*x*_, *p*_*y*_)	Global coordinates of the CM of the robot
α	Amplitude of sinusoidal motion pattern
ω	Frequency of sinusoidal motion pattern
δ	Phase shift between the joints with a sinusoidal motion pattern
ϕ_*o*_	Joint offset coordinate used for directional control
ϕ	Vector of joint angles ϕ_*i*_
ϕ^*^	Vector of reference joint angles $\phi_{i}^{*}$
Δ	Look-ahead distance
**p**_o_	Coordinates of the CM of the obstacle
θ_ref, pf_	Orientation of the robot during path following mode
θ_ref, oa_	Orientation of the robot during obstacle avoidance mode

Obstacle avoidance is by its very nature a safety feature which should be activated when necessary and otherwise not affect the behavior of the system. In this paper, the default mode of operation is straight line path following, although this objective may easily be replaced by another mode of operation to be combined with the proposed obstacle avoidance method. For more details, see section 2.3 and Moe and Pettersen (2016). To achieve a guidance system with a path following and an obstacle avoidance mode, we employ a guidance law from Kohl et al. (2017) which is suitable both for straight line and circular path following. The latter is applied for obstacle avoidance to encircle obstacles on the way.

Obstacles are avoided by ensuring that the USR always maintains a certain safe distance between itself and the obstacle. Thus, in our obstacle avoidance guidance system we propose to encircle an obstacle, whose center position is defined as ***P***_o_ = [*P*_ox_, *P* oy]_T_, with a virtual circle of radius *R*^s^. The circle center is anchored in the obstacle center, and the radius is chosen sufficiently large so that if the USR is outside or on *R*_s_, a collision will not occur. Therefore, *R*_s_ is referred to as the safe radius, and the formalized control objective of the obstacle avoidance is to ensure that the USR is always outside or on *R*_s_.

A variety of different path following control approaches for USRs without thrusters have been studied in previous literature (McIsaac and Ostrowski, 2003; Lapierre and Jouvencel, 2005; Alamir et al., 2007; Kelasidi et al., 2016a, 2017b). An introductory discussion comparing the different control approaches studied for underwater swimming robots can be found in (Kelasidi et al., 2016a, 2017b). In this paper, we present experimental results for the underwater snake robot Mamba with thrusters at the tail module, using the path following control approach described below.

The control approach consists of a path following guidance law responsible for producing the reference orientation θ_ref, pf_, the heading controller responsible for making the actual orientation θ track the reference orientation, and the control input *u*_*c*_ to the thrusters responsible to propel the robot forward. The reference orientation θ_ref, pf_ of the robot is calculated using the guidance law presented in (1), which for the straight line path following reduces to the well-known LOS guidance law. The LOS approach is based on a term guiding the vehicle in question along the desired path and another toward the path. The latter is reduced to zero when the vehicle is on the desired path and is commonly used both for marine vehicles (Børhaug and Pettersen, 2006; Breivik and Fossen, 2008) and USRs (Kelasidi et al., 2016a, 2017b).

In this paper, the reference path is aligned with the world *x*-axis. Therefore, the *y*-position of the USR *p*_*y*_ is defined as the path cross-track error for path following. The orientation of the robot was measured using the underwater camera positioning system as shown in Figure 3 by attaching reflective markers at the tail module of the robot. The heading controller (2) is used to generate the joint angle offset, ϕ_0_, which is sent to the robot via the CAN.

There are multiple possible definitions of the orientation of an USR (Kelasidi et al., 2016a; Kohl et al., 2016). In this paper, the orientation θ of the robot is defined as the orientation of the head angle θ: = θ_*N*_. In the experimental setup, the USR position and orientation is measured using the underwater positioning system in the lab (see Figure 3). The reference orientation is defined by the following guidance law (Kohl et al., 2017):

(1) $$\begin{array}{l} \theta_{\text{ref}}=\arctan\left(\frac{\mu_{y}}{\mu_{x}}\right),\\ \mu (p)=-\frac{dh_{p}^{T}}{\|dh_{p}{\|}^{2}}\left(k_{\text{tran}}h(p)\right)+\nu \left[\begin{array}{cc} 0 & 1\\ 1 & 0 \end{array}\right]dh_{p}^{T}\frac{k_{\text{along}}}{\left\|dh_{p}\right\|},\\ \nu =\{\begin{array}{ll} -1, & \text{circle }\;\text{counterclockwise}\\ +1, & \text{circle}\;\text{ clockwise} \end{array} \end{array}$$

Here, *h*(***p***) is a cost function that implicitly defines the reference path, $dh_{p}^{T}=\nabla h\left(p\right)$ is a vector that is normal to the level sets of *h*, *k*_tran_ is the transversal gain, and *k*_along_ the along-path gain. This reference angle is referred to as θ_ref, pf_ and is utilized as a reference for path following (see Figure 3).

Since $dh_{p}^{T}=\nabla h\left(p\right)$ is perpendicular to the level sets of *h*(·), the control law (1) can be intuitively described as follows. The reference velocity **μ**(***p***) is composed of two components: The first component is perpendicular to the level sets of *h*(·) and decreases the distance of the center of mass to the curve γ = *h*^−1^(0). The second component is tangent to the level sets of *h*(·) and regulates the velocity of the center of mass on the curve γ = *h*^−1^(0). The choice of ν enables us to choose the direction which the robot should follow around the obstacle.

Analogously, the angle θ_ref, oa_ obtained from (1) by using $h_{oa}\left(p\right)={\left(p_{x}-p_{\text{o}x}\right)}^{2}+{\left(p_{y}-p_{\text{o}y}\right)}^{2}-R_{\text{s}}^{2}$ is used as a reference for obstacle avoidance. In this case, the parameter ν controls the USR direction of motion, and is chosen such that the USR circumvents an obstacle by deviating as little as possible from the reference straight line path. Note that for this guidance scheme it is sufficient to know the position of the obstacles relative to the USR. However, in this paper we have calculated the obstacle world position because the obstacles are detected relative to the camera frame (attached to the USR head link), whereas the position of the USR is given as the CM.

When applied to a straight line, the guidance law (1) ensures that the USR converges to the reference path. However, for a circular path, the guidance law (1) ensures that the robot approaches the path and thereafter remains close to it with a constant offset outside the radius *R*_s_.

**Remark 1**. *Note that the offset can be made small by increasing *k*_tran_ or eliminated completely by adding integral action to the guidance law. However, in this paper we deliberately choose to employ a rather small *k*_tran_ and thus always keep the USR safely outside the circle, rather than ensure that it converges closer to the safe radius *R*_s_ and possibly overshoots*.

The final part of the guidance system is an algorithm which determines if path following or obstacle avoidance is the active mode. This is described in more detail in section 2.3.

In Sans-Muntadas et al. (2017) it is proposed to set the reference for each joint as

(2) $$\begin{array}{r} \phi_{i}^{*}\left(t\right)=\phi_{0}, \end{array}$$

i.e., to make each joint have the same value, providing an even curvature along the whole robot. This is different from Kohl et al. (2017) where undulations are used for propulsion, and the joint references include an additional sinusoidal term with a phase shift between the joints. Instead (1) ensures that the joints are used only for directional control, while the thrusters are used to propel the robot forward. In particular, Sans-Muntadas et al. (2017) has shown that by using (1) the robot managed to converge nicely toward and move along the desired path. Hence, in order to steer the thrusted USR to the reference orientation, the parameter ϕ_0_ is used to control the direction of the robot. To steer the orientation θ according to the guidance law (1), the following PD controller is used to define the joint angle offset (Kohl et al., 2017):

(3) $$\begin{array}{r} \phi_{0}=k_{p}\left(\theta_{\text{ref}}-\theta \right)+k_{d}\left({\dot{\theta }}_{\text{ref}}-\dot{\theta }\right) \end{array}$$

In the above equation, the control gains *k*_*p*_ and *k*_*d*_ are constant and positive. In addition, to ensure that the joint angle ϕ_*i*_ tracks the reference signal $\phi_{i}^{*}=\phi_{0}$, a low level P-controller is implemented in the microcontrollers inside each module of Mamba. Similarly, to assign a rotational speed to the thrusters, a corresponding low level controller is implemented to ensure that the two tail thrusters track the reference *u*_*c*_.

### 2.3. Set-Based Obstacle Avoidance

It is clear that tasks such as path following and obstacle avoidance are not necessarily compatible. If an obstacle is somewhere along the path, the USR either has to deviate from the path or collide. We therefore propose a switched control system with a path following and an obstacle avoidance mode. The default mode of operation, which is active as long as it will not lead to a collision, is path following. When the USR is close to an obstacle and path following will further decrease that distance, the system switches to collision avoidance mode.

The switched guidance system is based on recent results in set-based control (Moe et al., 2016). Here, a widely used kinematic control framework is extended to handle set-based tasks, which have a valid interval of values rather than en exact desired state. Obstacle avoidance may be described as such a task, where the distance between the USR and an obstacle should be kept within a certain interval. In particular, the valid interval is given by all positive numbers above the lower bound *R*_s_. However, the approach proposed in Moe et al. (2016) is applicable to redundant systems to fulfill several, compatible tasks simultaneously. Since the two control objectives, i.e., path following and obstacle avoidance, are not compatible, we therefore alter the approach according to Moe and Pettersen (2016) and Kohl et al. (2017) to switch between the two tasks, i.e., the two guidance laws θ_ref, pf_ and θ_ref, oa_ described in section 2.2.

For the switched system we introduce an additional circle, which is also anchored in the obstacle center *p*_o_, with a radius *R*_m_ > *R*_s_. The radius *R*_m_ is referred to as the *mode change radius*. Outside the mode change radius, the guidance system is always in path following mode. Inside *R*_m_, either mode may be active. If path following mode will not lead to the distance between the USR and the obstacle decreasing further, it is active. Otherwise, obstacle avoidance is activated, and the USR should converge toward the safe radius *R*_s_. The mode change radius must be chosen sufficiently large so that in case of a switch to obstacle avoidance mode, the USR converges to the safe radius without overshoot. This is partly achieved by tuning the obstacle avoidance guidance law such that the USR converges to an offset outside *R*_s_ rather than to the actual safe radius as described in the previous section. The desired switching behavior is captured by Algorithm 1, which is based on set-based theory described in Moe et al. (2016), Moe and Pettersen (2016), and Kohl et al. (2017).

**Remark 2**. *Note that a similar approach is applied in Kohl et al. (2017) for obstacle avoidance of snake robots without thrusters that is able to propel forward only by using undulatory gaits. Due to the oscillatory behavior of the swimming snake robots, the set-based approach must be more conservative to ensure that no part of the robot collides with the obstacle. For thrusted USRs in this paper, we exploit the fact that the thrusters ensure forward propulsion and the joints control the direction of motion by letting the robot safely curve around the obstacle*.

**Table:** **Algorithm 1:** The set-based switched guidance algorithm.

**Input**: σ, θ_ref, pf_, θ_o_
**if** σ ≥ *R*_m_ **then**
| θ_ref_ = θ_ref,pf_
**else if** $|\theta_{ref,pf}-\theta_{\text{o}}|\leq \frac{\pi }{2}$ **then**
| θ_ref_ = θ_ref, pf_
**else**
| θ_ref_ = θ_ref, oa_
**end**

**Figure 4:**
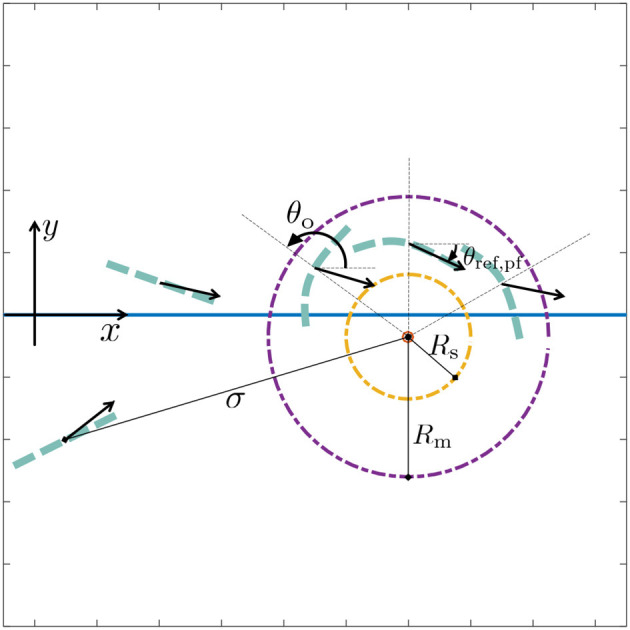
Obstacle avoidance parameters: the set-based task σ is defined as the distance between the obstacle center and the USR. Outside the mode change radius *R*_m_, the system is always in path following mode. The desired straight line path lies along the x-axis. The desired heading for path following is defined as θ_ref, pf_ and indicated by the black arrows for several USR positions and orientations. Inside *R*_m_, the system is in path following mode if it will lead to an increase in σ, i.e., when the angle between θ_ref, pf_ and θ_o_ is smaller than or equal to π/2. Otherwise, obstacle avoidance mode is active, in which case the desired heading is defined by θ_ref, oa_ and the USR should converge the and track the safe radius *R*_s_.

For the obstacle avoidance scenario described above, the obstacle avoidance task σ is defined as the distance between the USR CM and an obstacle. It has a valid interval *D* = [*R*_s_, ∞), and the input parameters are illustrated in Figure 4, where θ_ref, pf_ is the desired heading for path following and θ_o_ is the angular coordinate of the obstacle. Thus, as illustrated in Figure 4, path following will result in the distance between the USR and the obstacle increasing when the angle between θ_ref, pf_ and θ_o_ is less than π/2. In this case, path following is active also within the radius *R*_m_. Note that by using the CM of the USR when calculating σ, part of the USR is actually allowed within the safe radius *R*_s_. This must be accounted for by choosing a sufficiently large *R*_s_. Furthermore, the switching strategy in Algorithm 1 is completely general, and may be applied for any combination of guidance laws to achieve alternative desired behaviors such as target tracking, trajectory tracking or other path following schemes.

**Remark 3**. *Note that this method is valid for multiple obstacles given that said obstacles are not overlapping or moving. In these experiments, only one stationary obstacle was used due to the limited size of the test basin. Handling overlapping and moving obstacles is a topic for future work*.

### 2.4. Obstacle Detection

In this paper, we assume that the USR is to operate in some structured environment which we are free to influence, e.g., an underwater oil and gas structure. Hence, we presume that potential obstacles are marked with some sort of geometric shape that may be detected using a camera on the USR head and computer vision. Thus, obstacles of different sizes may be marked with different shapes. For unforeseen events such as debris another detection scheme must be applied. However, note that a set-based approach is still applicable for avoidance given estimation of obstacle position and velocity.

**Figure 5:**
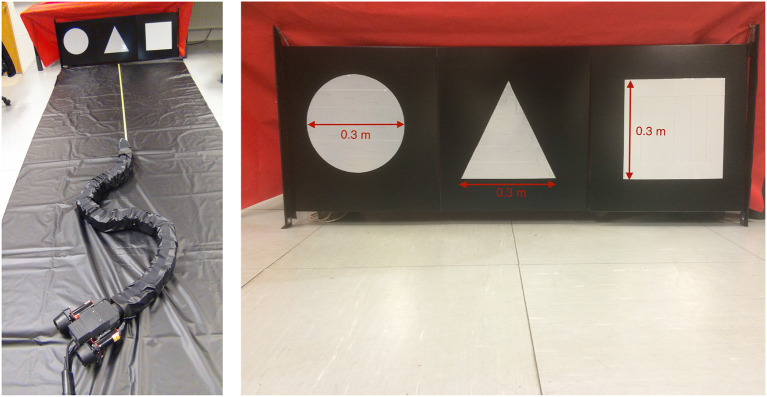
The robot Mamba with thrusters and the reflective markers representing obstacles.

For these experiments, we used the pinhole camera model (Medioni and Kang, 2004) to derive the equations applied in the implemented detection algorithm. Three geometric shapes with a known area *A*_r_ have been constructed using reflective tape, and these represent the obstacles in the experiments: a circle, a triangle and a square (see Figure 5). To avoid an obstacle by circumventing it as described in sections 2.2 and 2.3, the obstacle position in the world coordinate frame *p*_o_ must be known. Hence, the goal of the obstacle detection algorithm is to calculate this position.

**Figure 6:**
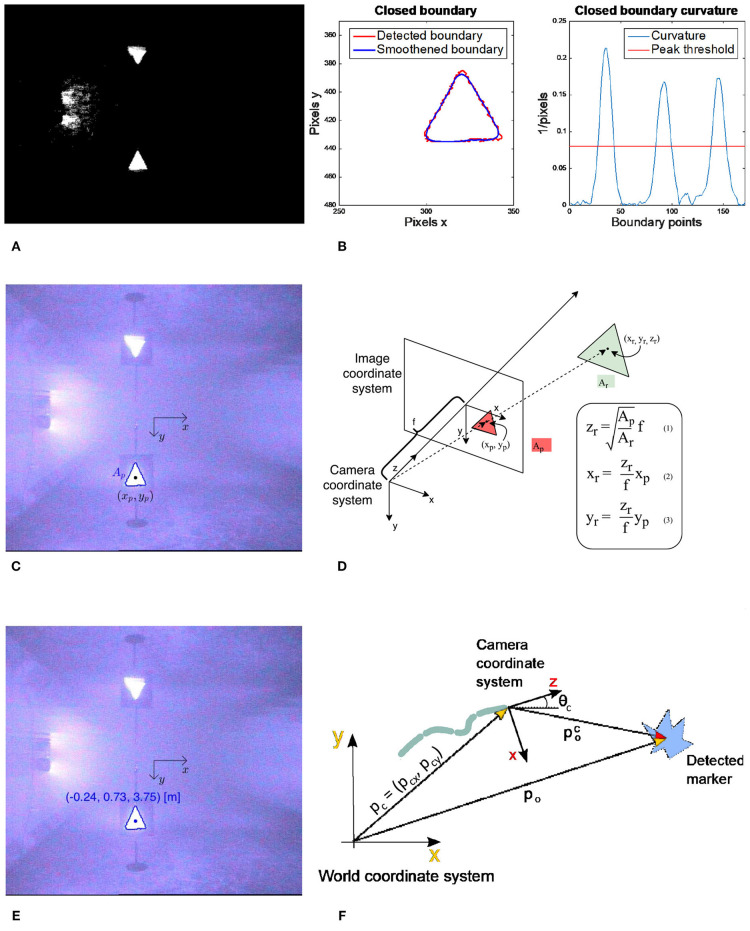
The 3D-position of a marker relative to the camera coordinate system ${\text{p}}_{\text{o}}^{\text{c}}={\left[x_{\text{r}},y_{\text{r}},z_{\text{r}}\right]}^{T}$ can be calculated using the focal length *f* of the camera, the area of the marker in the image *A*_P_ and reality *A*_r_ and the position of the marker in the image coordinate system (*x*_p_, *y*_p_). In this paper, the USR is moving in the plane, so the vertical component *y*_r_ can be ignored. **(A)** Convert input image to black and white. **(B)** Find closed boundaries, smoothen them, calculate curvature and count number of peaks to classify geometric shape. **(C)** Find the position (*x*_p_, *y*_p_) and the area of the shape *A*_p_ in the image coordinate system. **(D)** Use the pinhole camera model, camera focal length *f* and the actual area of the shape *A*_r_ to calculate the 3D-position of the detected shape relative to the camera coordinate frame. **(E)** The calculated position is referred to as ${\text{p}}_{\text{o}}^{\text{c}}=\left[x_{\text{r}},y_{\text{r}},z_{\text{r}}\right]$. **(F)** Use the USR camera position and orientation to calculate the detected position in the world coordinate frame ***p***_*o*_.

The obstacle detection algorithm is based on four main steps, which are illustrated in Figure 6: (1) Recognize and classify an obstacle marker as a triangle, square or circle, (2) find the position and area of the marker in the image *x*_p_, *y*_p_ and *A*_p_, (3) compare *A*_p_ to the actual area of the marker *A*_r_ and use the camera focal length *f* and the marker position in the image to calculate the 3D obstacle position relative to the camera ${\text{p}}_{\text{o}}^{\text{c}}$, and (4) find the obstacle position relative to the world coordinate frame *p*_o_ by rotating and transltaing about the camera frame orientation and position. The detailed implementation can be found in Algorithm 2.

**Remark 4**. *Note that the equations in Figure 6D are based on the assumption that the obstacle marker is oriented parallel to the camera coordinate system *xy*-plane, i.e., that all the corners of the triangle have the same *z*-coordinate. This assumption is not satisfied if the camera is looking at a marker at an angle. However, due to the relatively small size of the markers, the potential difference in the *z*-coordinate of the corners is limited and small compared to the distance at which it is necessary to observe them to successfully avoid the obstacle. Thus, this assumption is a valid approximation and will result in a limited error in the calculated position*.

## 3. Experimental Results

In this section, we discuss the obtained experimental results for the proposed path following control strategy (section 3.1) and the obstacle detection and avoidance concept (sections 3.2–3.3) described in previous section using the thrusted USR Mamba.

### 3.1. Straight Line Path Following

In all experiments the joint angles of the robot were set to zero, whereas the initial orientation, θ(0), the position of the CM of the robot along the *y* axis, *p*_*y*_, the proportional control gain, *k*_*p*_, the look-ahead distance, Δ, and the control input to the thrusters, *u*_*c*_, are displayed in Table 2 for each trial. The average power consumption is calculated by using the following expression

(4) $$\begin{array}{r} P_{\text{avg}}=VI_{\text{avg}}, \end{array}$$

where *V* = 35 [V] and *I*_avg_ [A] is the average current that is measured using the high performance industrial logging multimeter FLUKE 289. In addition, the average forward velocity for each experimental trial was calculated as

(5) $$\begin{array}{r} \bar{\upsilon }=\left(\sqrt{{\left(p_{\text{stop},x}-p_{\text{start},x}\right)}^{2}+{\left(p_{\text{stop},y}-p_{\text{start},y}\right)}^{2}}\right)/\left(t_{\text{stop}}-t_{\text{start}}\right), \end{array}$$

where **p**_start_ and **p**_stop_ represent the initial and the final points of the distance traveled in the time interval *t*_stop_ − *t*_start_. The control gain *k*_*d*_ was set to zero for the experimental results presented for the straight line path following control approach. In addition, the joint offset ϕ_0_ has been saturated at ±20° to ensure that the physical limitation of the robot joint angles is not exceeded.

**Table 2:** The average forward velocity and power consumption for the path following case studies using the underwater snake robot mamba with thrusters.

	***u*_*c*_**	***F*_*t*_ [N]**	**Δ [m]**	***k*_*p*_**	**θ(0) [deg]**	***p*_*y*_(0) [m]**	**$\bar{\upsilon }$ [m/s]**	**P_avg_ [W]**
Path 1	60	2.4362	1	0.18	-82.70	0.8905	0.2468	63.8400
Path 2	60	2.4362	1	0.09	-26.20	0.9544	0.2265	53.3855
Path 3	60	2.4362	1	0.09	21.4	1.7991	0.2167	58.4885
Path 4	60	2.4362	1	0.13	-2.70	1.1570	0.2240	45.8850

Previous experimental results for path following of underwater snake robots using the body undulation for both propulsion and directional control, showed that the robot was able to reach and follow the path using the LOS guidance law (Kelasidi et al., 2016a, 2017b). However, the use of an oscillatory gait pattern causes steady state oscillations about zero for the cross-track error and the orientation, which is expected since it is difficult to achieve a purely non-oscillating motion for the CM and the orientation of underwater swimming snake robots (Kelasidi et al., 2016a, 2017b). These oscillations can be restrictive for several applications in subsea environment, such as for instance docking (Sans-Muntadas et al., 2017).

**Figure 7:**
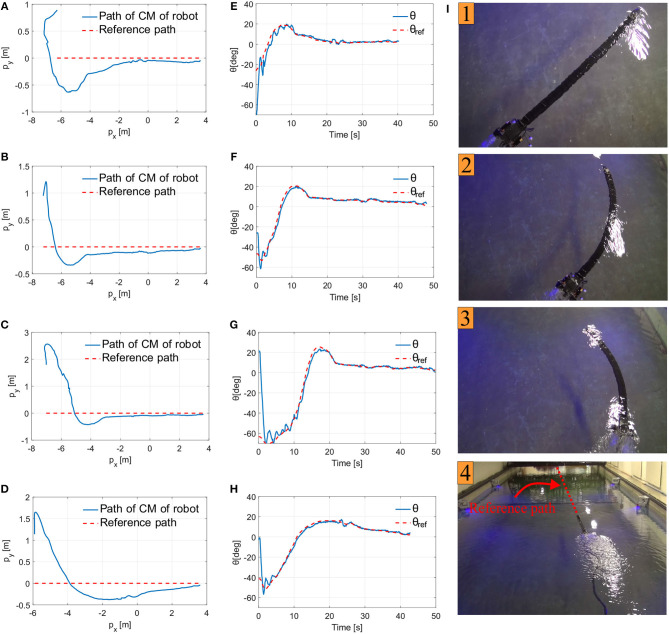
Experimental results path following. **(A)** Center of mass position for *u*_*c*_ = 60, *k*_*p*_ = 1, *k*_*d*_ = 0, Δ = 180cm and θ = −82.7^o^. **(B)** Center of mass position for *u*_*c*_ = 60, *k*_*p*_ = 1, *k*_*d*_ = 0, Δ = 90cm and θ = −26.2^o^. **(C)** Center of mass position for *u*_*c*_ = 60, *k*_*p*_ = 1, *k*_*d*_ = 0, Δ = 90cm and θ = 21.4^o^. **(D)** Center of mass position for *u*_*c*_ = 60, *k*_*p*_ = 1, *k*_*d*_ = 0, Δ = 130cm and θ = 2.7^o^. **(E)** The orientation of the robot for case **(A)**. **(F)** The orientation of the robot for case **(B)**. **(G)** The orientation of the robot for case **(C)**. **(H)** The orientation of the robot for case **(D)**. **(I)** The motion of the underwater snake robot Mamba with thrusters during the path following for the experimental results presented in **(C,G)**, where the *red line* indicates the reference path.

Experimental results for four different path following trials of Mamba with thrusters are presented in Figure 7, see also the Supplementary Videos. As Figure 7 illustrates, the robot manages to converge to and follow the desired path for all the investigated cases. Furthermore, the reference orientation is tracked without oscillations. The overshoot and the initial rapid change on the orientation shown in Figure 7 as the snake robot converges to the path is a result of the tuning, and in particular the choice of the look-ahead distance Δ. The larger the choice of Δ is, the smaller the overshoot will be, and the slower the convergence rate will be. The choice of Δ is thus a trade-off between convergence and the overshoot (Kelasidi et al., 2017b). The small steady state error in cross-track error may be a result of several factors, such as the possible misalignment of the two thrusters used at the tail module of the robot, measurement errors from the different sensors used during the experiments, and the forces on the robot due to the use of the tether. In the future, more advanced heading control approaches can be investigated to remove this small error, for instance by including integral action (Caharija et al., 2012). In addition to the convergence to the straight line path, we obtained results regarding the achieved forward velocity and the power consumption for all the investigated trials, and these are shown in Table 2. The achieved velocity is very similar in all trials, which is expected since the same control input value for the thrusters was used for all the investigated cases. However, the average power consumption varies for the different investigated path following case studies as shown in Table 2. This is reasonable since the power consumption is related not only to the trusters, but also to the joint modules. The actuation of the directional control and thereby the joint motion for each trial depends on the initial heading and distance from the path, which varies in the different trials.

### 3.2. Obstacle Detection

For the experiments presented in this paper, the obstacle detection algorithm was run off-line and the detected position was added manually to the switched guidance and control system described in section 2.3. However, the algorithm also has the potential to be fully autonomous as part of the online control system; the detection algorithm is sufficiently fast that runtime will not be a concern in an online implementation. The implementation of the necessary communication and control structure required to achieve this is a topic of future work. However, in the presented results the obstacle detection algorithm was run and the detected position added to the control system in one operation without removing the robot from the pool or turning it off.

**Figure 8:**
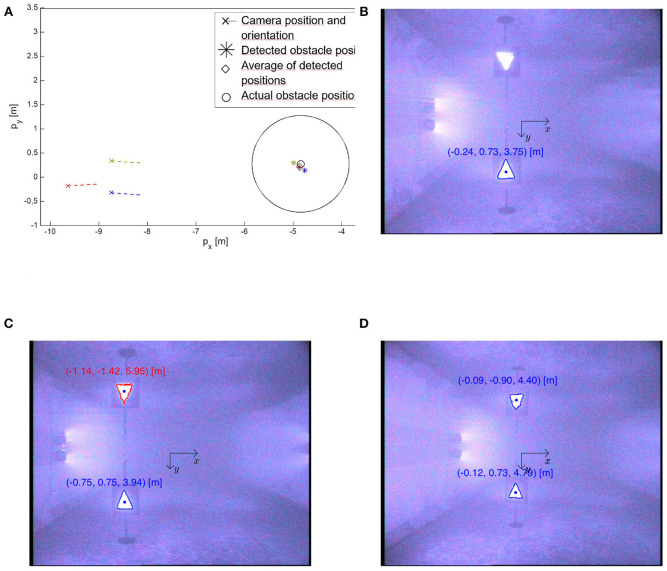
The obstacle detection algorithm described in Figure 6 was run three times for different USR positions and orientation shown as magenta, blue and red, respectively. For the two latter cases, the algorithm also detects the distorted surface reflection of the markers and classifies it as a square and a triangle, respectively. For a true underwater applications this phenomena will not occur, and these fake detections are easily disregarded by observing that their *y*-coordinate is negative, i.e., they are above the surface. **(A)** The average detected position ♢ was 7 cm from the actual obstacle position ○. **(B)** The detected obstacle position relative to the camera ${\text{p}}_{\text{o}}^{\text{c}}$ corresponding to magenta in **(A)**. **(C)** The detected obstacle position relative to the camera ${\text{p}}_{\text{o}}^{\text{c}}$ corresponding to blue in **(A)**. **(D)** The detected obstacle position relative to the camera ${\text{p}}_{\text{o}}^{\text{c}}$ corresponding to red in **(A)**.

**Figure 9:**
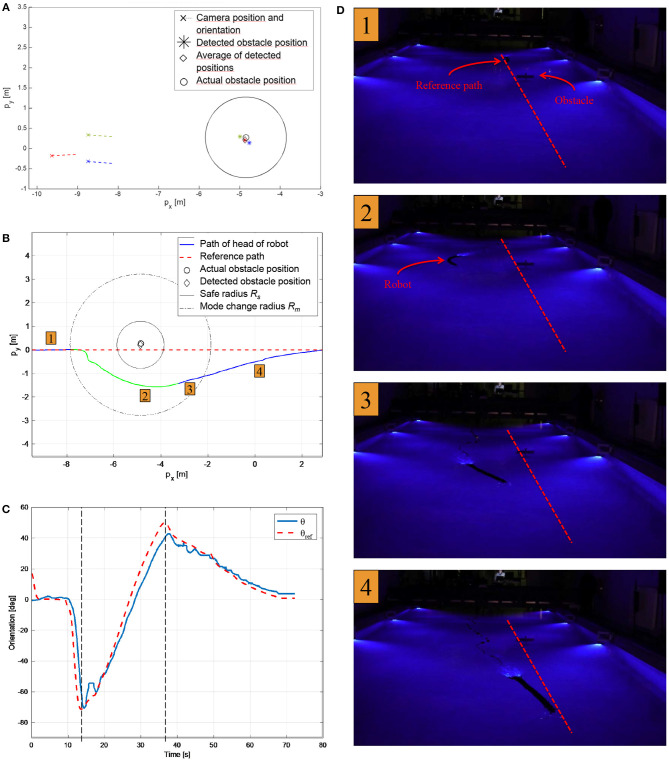
Experimental results for path following with detected obstacle position and obstacle avoidance. **(A)** The obstacle detection algorithm described in Figure 6 was run three times for different USR positions and orientation. The average detected position ♢ was approximately 0.07 m from the actual obstacle position ○. **(B)** The path of the USR as it follows the path (blue), switches to obstacle avoidance and circumvents the detected obstacle (green) and finally switches back to path following (blue). **(C)** The actual and desired orientation of the robot. The control system switches from path following to obstacle avoidance at ≈ *t* = 13 s and back again at ≈ *t* = 37 s. **(D)** The motion of the underwater snake Mamba with thrusters during the obstacle detection and avoidance experiments. Corresponding positions are indicated in **(B)**.

To detect the obstacle position, the detection algorithm described in Figure 6 was run three times for different camera positions and orientations, see Figures 8, 9A. Note that when testing the obstacle detection scheme, the available USR positions and orientations where the obstacle marker was in the camera frame were limited by the pool size and the Qualisys tracking system. The average of the three detected positions *p*_o_ was then inserted into the control system and used for the remainder of the experiments. To quantify the accuracy of the algorithm, the actual position of the obstacle was measured using the Qualisys tracking system, and the final detected position *p*_o_ was approximately 0.07 m from the actual obstacle position, which corresponds to 7% of the safe radius *R*_s_ = 1 m and 3.9% of the total length of the robot. This result is sufficiently accurate to safely use for the obstacle avoidance scheme, thereby confirming that the proposed detection approach is highly applicable. Note that to achieve a sufficiently good visual to detect and classify the reflective markers and to simulate a subsea environment as closely as possible, all lights were turned off during the experiments with the exception of the lights on the camera of the USR and the Qualisys tracking system.

**Table:**
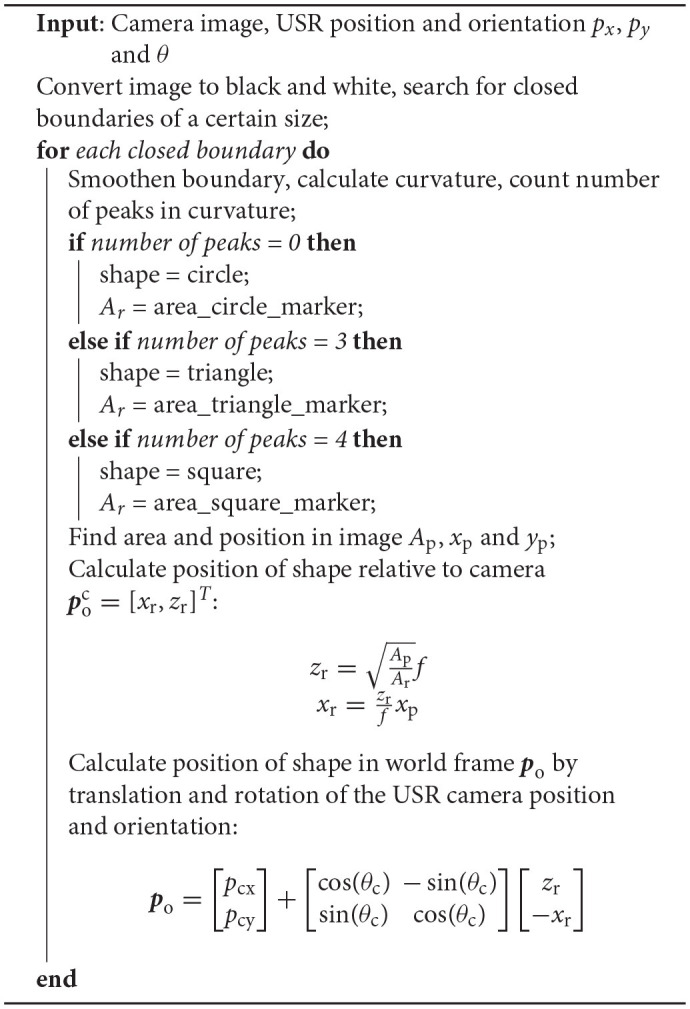
**Algorithm 2:** Obstacle detection algorithm.

### 3.3. Obstacle Avoidance

The experimental setup for path following and obstacle avoidance is identical to the one described in the previous section, and experimental results are shown in Figures 9B–D. A recording of the experiment can be seen in the Supplementary Videos. The USR initial position is on the reference path, and the initial mode is path following. Once the USR enters the mode change radius *R*_m_ = 3 m, it is evident that continued path following will result in the USR getting closer to the obstacle. Hence, obstacle avoidance is activated, and the robot circumvents the obstacle by turning and attempting to stay outside the safe radius *R*_s_ = 1 m. According to the theory described in section 2.2, the USR converges to a constant offset of the safe radius, which could be made smaller by a different choice of control gains. However, for this application it is crucial to avoid overshoot into *R*_s_, and thus a larger offset is preferable. Furthermore, the position of the USR is defined by the CM, which also requires a more conservative approach since part of the USR will in fact be allowed to enter the safe radius *R*_*s*_ and must be able to do so safely. Finally, the physical obstacle in the pool partly blocked the camera tracking system, making it infeasible with the available experimental setup to attempt less conservative approaches which would exploit the flexibility of the USR better. Note that the robot circumvents the obstacle by choosing the direction along the circle that ensures the shortest path as discussed in section 2.2.

As the USR circumvents the obstacle, path following will once again ensure that the robot moves away from the obstacle. Path following is then reactivated and the robot converges back to the path. This can be seen in Figure 9B. Figure 9C displays the reference orientation provided by the switched guidance system and the actual orientation of the USR. The implemented PD-controller ensures that the reference is tracked in a sufficiently accurate manner. Note that the switched guidance system described in Algorithm 1 results in abrupt changes in the reference orientation when the system switches between path following and obstacle avoidance. To provide the control system with a feasible reference signal, a hyperbolic smoothing function is implemented to ensure a continuous reference signal after a switch (Kohl et al., 2017). In addition, the commanded joint offset, ϕ_0_, is filtered with a first-order low-pass filter before it enters the low-level controller.

Figure 9D displays images from the experiment. The USR clearly circumvents the obstacle on a circular path before converging back to the reference path. The chosen control parameters for the implementation are as follows:

1. Path following guidance law θ_ref, pf_: *k*_tran_ = 0.1, *k*_along_ = 0.15 (corresponding to a look-ahead distance Δ = 1.5 m)
2. Obstacle avoidance guidance law θ_ref, oa_: *k*_tran_ = 0.02, *k*_along_ = 0.15
3. Controller ϕ_o_: *k*_*p*_ = 0.42, *k*_*d*_ = 0.03
4. Smoothing function:
  $$\begin{array}{l} \alpha \left(t,t_{\text{last switch}}\right)=\frac{1}{2}\left(\tanh\left(\alpha_{1}\left(t-t_{\text{last switch}}-\alpha_{2}\right)+1\right)\right),\\ \;\alpha_{1}=1.2,\alpha_{2}=1.6 \end{array}$$

## 4. Conclusions

USRs have a multitude of essential qualities for autonomous underwater operations, such as efficient locomotion, flexible bodies and the possibility to perform intervention tasks. These highly versatile robots may be equipped with different modules such as thrusters or fins, and are applicable for a variety of tasks within several fields of research.

In this paper, we present a guidance and control system to ensure path following and obstacle avoidance of a USR with thrusters, in addition to a computer vision algorithm to detect and calculate the position of potential obstacles. Based on preliminary results to ensure energy efficient motion and high velocity, the USR motion relies on thrusters for forward propulsion, whereas directional control is achieved through the joints of the body. The proposed methods are all experimentally verified for the first time, using the thrusted USR Mamba for the first time. Future work includes extending the proposed guidance and control approach to 3D in order to be able to investigate path following and obstacle avoidance of USR with thrusters in 3D.

## Author Contributions

All authors listed have made a substantial, direct and intellectual contribution to the work, and approved it for publication.

### Conflict of Interest Statement

The authors declare that the research was conducted in the absence of any commercial or financial relationships that could be construed as a potential conflict of interest.

## Supplementary Material

Supplementary Video 1 Obstacle avoidance.

Supplementary Video 2 Path following thrusted Mamba.
